# Treatment of stage 4s neuroblastoma – report of 10 years' experience of the French Society of Paediatric Oncology (SFOP)

**DOI:** 10.1038/sj.bjc.6601154

**Published:** 2003-07-29

**Authors:** G Schleiermacher, H Rubie, O Hartmann, C Bergeron, P Chastagner, F Mechinaud, J Michon

**Affiliations:** 1 Département de Pédiatrie, Institut Curie, 26 rued'Ulm, 75248 Paris Cedex 05, France; 2 INSERM U509 Pathologie Moléculaire des Cancers, Institut Curie, Paris, France; 3 Unité d'Hémato-oncologie Pédiatrique, Hôpital des Enfants, Toulouse, France; 4 Département d'Oncologie Pédiatrique, Institut Gustave Roussy, Villejuif, France; 5 Département de Pédiatrie, Centre Leon Bérard, Lyon, France; 6 Unité d'Hémato-oncologie Pédiatrique, Hôpital des Enfants, Nancy, France; 7 Unité d'Hémato-oncologie Pédiatrique, Hôpital de la Mère et l'Enfant, Nantes, France

**Keywords:** neuroblastoma, stage 4s, prognosis, treatment

## Abstract

Stage 4s neuroblastoma (NB) is usually associated with a favourable outcome, despite a large tumour burden, as spontaneous regression frequently occurs. However, in some infants rapid disease progression can be observed with severe functional impairment. Thus, for all patients the potential risks of cytotoxic therapy must be weighed against the benefits of early medical intervention. We have retrospectively reviewed the charts of 94 infants treated for stage 4s NB in centres of the French Society of Paediatric Oncology between 1990 and 2000, and describe the different first-line treatment approaches that were, successively, liver irradiation, chemotherapy using a cyclophosphamide–vincristine regimen, and chemotherapy using a carboplatin–etoposide regimen. The overall survival was 88% (±7.6%), with a mean follow-up of 64 months. Elevated serum neuron-specific enolase (>100 nmol ml^−1^), ferritin (>280 ng ml^−1^) and urinary dopamine levels (>2500 nmol mmol^−1^ creatinine) were associated with a poor outcome, as were the genetic markers N-myc amplification and chromosome 1p deletion (*P*<0.0005 and *P*=0.0016, respectively). Patients who required medical intervention at diagnosis fared worse than those who received supportive treatment only (*P*<0.005). The clinical evolution observed with the different successive treatment approaches suggests that if infants do require therapy, the prompt initiation of a more intensive regimen such as carboplatin–etoposide may be more beneficial.

Neuroblastoma (NB) is the most common extracranial solid tumour of childhood. Over one-half of patients present with metastatic disease at diagnosis. In the case of widespread disease with bone and bone marrow metastases in children over 1 year of age, a dismal outcome is observed despite intensive multimodal therapy. Conversely, a small group of patients with disseminated disease have a good prognosis. This is a salient feature of NB 4s, a ‘special’ stage of disseminated NB first described in 1971 by Evans *et al* (1971) as stage IVs and referring to a small primary tumour with disease dissemination restricted to the liver, skin and/or bone marrow. Other staging systems have been described in the past for this disease. However, more recently, in order to create a uniform classification system, the International Neuroblastoma Staging System (INSS) has been implemented and is now used universally (Brodeur *et al*, 1988, 1993). The INSS criteria for stage 4s NB are similar to those of the original Evans' classification, but restrict this diagnosis to children of less than 1 year at diagnosis and with less than 10% of bone marrow involvement (Brodeur *et al*, 1993). Stage 4s NB represents approximately 7–10% of all NB cases. Its hallmark is the possibility of spontaneous regression despite a large tumour burden, and an overall good prognosis (Pritchard and Hickman, 1994; Matthay 1998; Hero *et al*, 2000). Indeed, large retrospective studies have demonstrated disease-free survival rates of 57–87% (Evans *et al*, 1980; Mancini *et al*, 1984; Katzenstein *et al*, 1998). More recently, a large prospective study has demonstrated a 5-year overall survival (OS) of 92% (Nickerson *et al*, 2000). Despite the fact that most infants have a good outcome with little or no treatment, neonates especially are at risk due to respiratory, renal, gastrointestinal impairment or coagulation disorders caused by massive hepatomegaly. Thus, chemotherapy and/or radiation therapy have been administered to those infants who present with progressive disease (PD) and life-threatening symptoms. However, in all cases, the potential benefits of early cytotoxic treatment need to be weighed against the possible adverse early and late effects of a medical intervention, and the decision of who, when, how and for how long to treat remains controversial (Hartwig *et al*, 1999). Objective criteria with a scoring scale indicating the degree of organ compromise have been described as a basis for treatment decisions (Hsu *et al*, 1996), but this scoring system is not universally employed. Furthermore, several well-defined parameters have been associated with a poor prognosis in stage 4s NB. These include age less than 2 months at diagnosis, biological markers such as elevated serum lactate dehydrogenase (LDH), ferritin or neuron-specific enolase (NSE) levels, histopathological as well as genetic markers such as N-myc amplification (NMA) and tumour diploidy (Seeger *et al*, 1985; Look *et al*, 1991; Evans *et al*, 1996; Katzenstein *et al*, 1998). Chromosome 1p deletion has also been suggested to be associated with a poor outcome in NB (Caron, 1995). When indicated, a wide range of different first-line treatment approaches have been used, ranging from liver radiotherapy as first-line treatment to more or less intensive chemotherapy regimens, but the heterogeneity of these approaches hamper the comparison of treatment results.

We now report on the French Society of Paediatric Oncology's experience with stage 4s NB during the last decade. We describe the clinical evolution of this disease in a series of 94 patients who were treated according to different successive therapeutic strategies. We furthermore aim to define clinical and biological prognostic factors in this series of patients.

## PATIENTS AND METHODS

Between 1990 and 2000, all infants with NB treated in centres of the SFOP (French Society of Paediatric Oncology) and aged less than 1 year at diagnosis were registered in the NBL90 study, with data provided by the treating physician. Stage 4s NB was defined according to the revised INSS criteria, on the basis of the provided data, including infants with small primary tumours and metastasis limited to skin, liver and bone marrow, without bone involvement (Brodeur *et al*, 1988; Brodeur *et al*, 1993). Those patients who did not undergo surgical evaluation of the primary tumour required by the INSS criteria were classified according to clinical and radiological findings. Infants with stage 4s NB were included in this study if clinical and follow-up data were available for retrospective data analysis. Patients with liver involvement due to contiguous tumour spread from a large intra-abdominal primary were excluded.

The diagnosis of NB was confirmed histologically either by surgical biopsies or more frequently cytologically by percutaneous fine-needle aspirates. Staging procedures at diagnosis involved evaluation of the extent of the primary tumour by ultrasound, computed tomography or magnetic resonance imaging, and level of urinary catecholamine metabolites on 24-h urine samples, standardised on urinary creatinine excretion. In order to evaluate metastatic spread, appropriate skeletal radiological imaging and MIBG scanning were performed, as well as bone marrow aspirates and bone marrow biopsies whenever possible. Local laboratories determined serum LDH, ferritin and NSE levels at diagnosis. N-myc copy number was determined in one of three SFOP reference laboratories by Southern blot analysis, dot blot, or semiquantitative polymerase chain reaction. Chromosome 1p status was determined by fluorescence *in situ* hybridisation or by search for loss-of-heterozygosity (LOH) at polymorphic loci.

Treatment consisted of supportive care for all infants. Life-threatening symptoms with respiratory or cardiovascular compromise were indications for treatment by either radiation therapy or chemotherapy. In some instances, very large but stable tumours were also treated due to threatening decompensation. The decision regarding which treatment plan to follow was taken by the treating physician according to the recommendations of the SFOP. Radiation therapy consisted of hepatic irradiation at a total dose of 4.5 Gy administered in three fractions of 1.5 Gy on three consecutive days. Different chemotherapy regimens were used. Until 1996, first-line chemotherapy consisted of the CO regimen (cyclophosphamide, 5 mg kg^−1^ day^−1^, days 1–3 and vincristine, 0.05 mg kg^−1^ day^−1^, day 1), administered every 2 weeks for a total of up to nine courses. More intensive chemotherapy was delivered according to the CE regimen (carboplatin, 6.6 mg kg^−1^ day^−1^, days 1–3 and etoposide, 5 mg kg^−1^ day^−1^, days 1–3), repeated after 21 days, which was used as a second-line chemotherapy before and as a first-line chemotherapy regimen after 1996. Other second-line treatment schedules consisted of the CADO regimen (cyclophosphamide 10 mg kg^−1^ day^−1^, days 1–5, vincristine 0.05 mg kg^−1^ day^−1^, days 1 and 5, and doxorubicin 2 mg kg^−1^ day^−1^, day 5) or the etoposide–cisplatin regimen (etoposide 3.3 mg kg^−1^ day^−1^, days 1–5 and cisplatin 1.3 mg kg^−1^ day^−1^, days 1–5). For the two latter schedules, doses were reduced to 2/3 where body weight was less than 10 kg. Five patients received high-dose chemotherapy (Busulphan–melphalan, BCNU-VM26-carboplatin, or carboplatin–melphalan) followed by autologous bone marrow or peripheral stem cell rescue. All chemotherapy courses were only commenced when the absolute neutrophil count was ⩾0.5 × 10^6^ ml^−1^.

Complete remission was defined as an absence of clinical or radiological evidence of disease and normalisation of urinary catecholamines.

### Statistical analysis

OS was calculated from the date of diagnosis. Survival rates were estimated according to the Kaplan–Meier method, and survival rates between subgroups of patients were compared using the log-rank test. Owing to the small number of events in this cohort of patients, and because all biologic parameters could not be determined for every patient, only univariate analyses were performed.

## RESULTS

### Population

A total of 97 patients were treated for stage 4s NB in centres of the SFOP between 1990 and 2000. For three patients, insufficient clinical data were available, and they were excluded from this study. Thus, 94 infants met the criteria for inclusion in this study, with a mean follow-up (FU) of 64 months (range 17–133 months). There were 50 boys and 44 girls, with a mean age at diagnosis of 66 days (range 0–261 days). The localisation of the primary tumour was most frequently abdominal (82 out of 94, 85%), with 13 patients presenting with bilateral adrenal disease, and two patients presenting with abdominal dumbbell tumours. Primary cervical and thoracic tumours were less frequent, and in five cases, the primary tumour could not be localised (Table 1

**Table 1:** Localisation of the primary tumour and of metastasis in 94 infants with stage 4s neuroblastoma

		**Metastasis**				
**Localisation of the primary tumour**		**Liver**	**Bone marrow**	**Lymph nodes**	**Skin**	**Other**
Abdominal	67 (37 left, 30 right)	63	19/60 evaluated	6	9	1
Abdominal (bilateral)	13	12	4/12 evaluated	1	3	0
Abdominal (dumbbell)	2	0	1	0	1	0
Thoracic	4	4	2	0	0	0
Cervical	2	2	1	0	1	0
Cervical and thoracic	1	1	0	0	0	0
Unknown	5	5	0	0	2	0
						
Total	94	87	27/86 evaluated	7	16	1

). Most tumours showed hepatic dissemination (87 out of 94, 92%), with bone marrow and skin metastasis occurring much less frequently.

### Medical treatment

**Figure 1:**
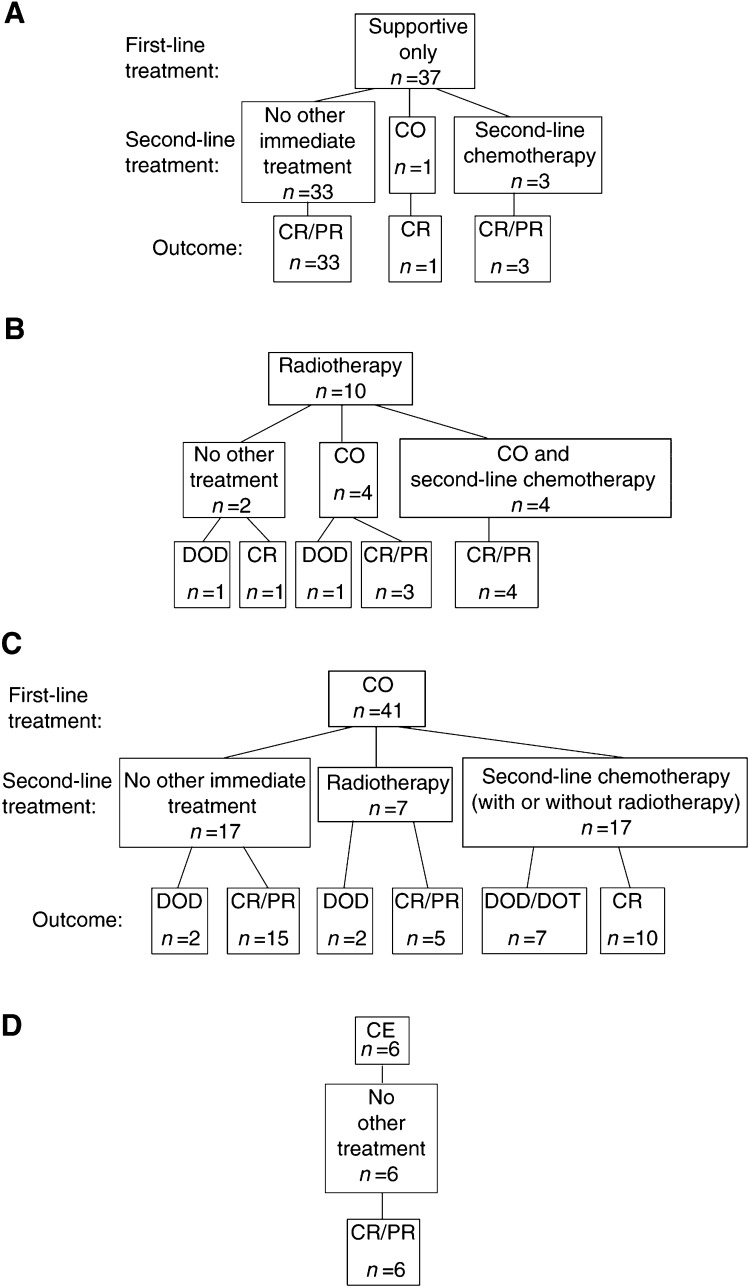
First- and second-line treatment of 94 patients with stage 4 s neuroblastoma, and outcome. CO-cyclophosphamide–vincristine regimen; CE-carboplatin–etoposide regimen. DOD/DOT-dead of disease/dead of toxicity. CR/PR-complete/partial remission.

According to the therapeutic strategy, infants without life-threatening symptoms were observed clinically and received supportive treatment only. A total of 37 out of 94 patients (40%) did not present with life-threatening clinical symptoms initially and thus did not require medical intervention. However, four of these patients presented with disease progression later and received chemotherapy at the time of progression. Two others experienced disease progression without worsening of clinical symptoms, and showed subsequent spontaneous regression. All these patients are alive, 31 are in complete (CR) and six in partial remission (PR), with a mean FU of 57 months. However, one patient who did not receive any medical treatment for stage 4s NB is currently being treated for acute lymphoblastic leukaemia which was diagnosed 25 months later (Figure 1

).

In all, 57 infants (60%) presented with life-threatening symptoms and required active treatment. Between 1990 and 2000, three different therapeutic strategies were recommended consecutively as first-line treatment. These consisted of liver radiotherapy (1990–1993), of the CO regimen (1994–1996), and of the CE regimen (1997–2000). In some cases, the CO regimen was also used as first-line treatment before 1993 and after 1997 (Table 2

**Table 2:** Number of patients treated according to different treatment strategies between 1990 and 2000

			**First-line treatment**		
**Time period**	**Patients (total)**	**Supportive treatment only**	**Liver radiotherapy**	**CO**	**CE**
1990–1993	41	12	10	19	0
1994–1996	30	15	0	15	0
1997–2000	23	10	0	7	6
					
	94	37	10	41	6

CO=cyclophosphamide–vincristine; CE=carboplatin–etoposide.

).

The therapeutic strategy recommended between 1990 and 1993 consisted of liver radiotherapy first followed by chemotherapy should radiotherapy prove to be insufficient. In total, 10 infants were treated with radiotherapy first. Among them, radiotherapy was the only treatment for two, one of whom relapsed 8 months after diagnosis and died. Eight others received chemotherapy in addition to liver irradiation (CO±second-line chemotherapy), and one of these patients also received high-dose chemotherapy followed by autologous stem cell rescue for hepatic and pulmonary relapse. One of these patients died early of PD, whereas the others are in CR (six patients (pts) or PR (one pt) with a mean FU of 98 months.

After 1993, a new treatment strategy was adopted which now consisted of chemotherapy with the CO regimen as first-line treatment. In total, 41 infants were treated with this schedule over the whole 10-year period. The mean delay between diagnosis and the first course of CO was 21 days (range 0–51 days), and a mean of four courses were administered (range 1–9 courses), either until tumour regression occurred or until a switch to second-line treatment. Only 17 of these patients (41%) did not receive second-line therapy, two of whom died due to disease progression, whereas 15 are in CR (14 pts) or PR (1 pt) with a mean FU of 80 months. Seven patients received liver radiotherapy following first-line treatment with CO. Two of these patients have died of PD, whereas five are alive in CR (four pts) or PR (one pt), with a mean FU of 68 months. Finally, following a first-line treatment regimen by CO 17 patients received second-line chemotherapy, followed by high-dose chemotherapy with autologous stem cell rescue in four cases, in one case due to the presence of amplification of the N-myc oncogene (NMA), and in three cases for progression to stage 4 disease. Seven of these patients have died due to PD (six cases) or toxicity (one case), whereas 10 are alive in CR (nine pts) or PR (one pt), with a mean FU of 58 months.

Altogether, first-line treatment including liver irradiation and/or the CO regimen was effective for only 24 out of 51 infants (45%), whereas second-line chemotherapy was needed in 21 cases.

In most cases, second-line chemotherapy consisted of two courses of the CE regimen. In all, 19 patients received this regimen as second-line treatment. The mean delay between diagnosis and the first CE course was 155 days (range 23–432 days), and the mean age at the first course of CE was 155 days (range 50–490 days). After two courses of CE, 2 out of 19 patients did not show any response to this treatment and died, whereas a partial response was observed in 17 patients. They either received two subsequent courses of the same regimen (eight pts) or two courses of the CADO regimen (nine pts). Seven of these patients relapsed later, and five of these patients have died (one from toxicity, four from disease), whereas 12 are alive in PR (two pts) or CR (10 pts).

After 1996, the CE regimen was recommended as firstline treatment for all infants presenting with life-threatening symptoms and was given to six patients. The mean delay between diagnosis and chemotherapy was 15 days (5–65 days). Tumour response was observed after two courses of CE in all six patients, and none required any further treatment. All are alive in CR (five pts) or PR (one pt) with a mean FU of 26 months (range 17–37 months).

The toxicity of the CE regimen was mainly haematological and always manageable, with a mean delay between the first and second course of 21 days (16–25 days). Neutropenia was observed after four courses, and anaemia requiring red cell transfusion after two courses. Two patients presented with minor infectious complications. No other complications were observed with this regimen.

### Outcome

The 3-year OS of this population was 88% (±7.6%) (Table 3

**Table 3:** OS and prognostic factors in 94 infants with stage 4s neuroblastoma

**Parameter**		***N***	**3-year OS (%) (±2 s.e.)**	***P*** **(log-rank)**
Total population		94	88 (±7.6)	
Age at diagnosis	⩽60 days	51	83 (±12.2)	0.18
	>60 days	43	93 (±8)	
	⩽90 days	62	82 (±11.4)	
	>90 days	32	96 (±6)	<0.06
	⩽120 days	73	84 (±9.8)	
	>120 days	21	100	0.06
				
Primary tumour	Abdominal	82	89 (±7.8)	
	Extra-abdominal	7	71 (±31.2)	0.12
				
Metastasis	No liver metastasis	7	100	
	Liver metastasis	87	87 (±8.2)	0.33
	No cutaneous metastasis	78	88 (±8.2)	
	Cutaneous metastasis	16	87 (±16.8)	0.87
	No bone marrow metastasis	59	86 (±10)	
	Bone marrow metastasis	27	88 (±13)	0.75
				
Medical treatment	Supportive	37	100	
	chemotherapy and/or radiotherapy	57	80 (±11.8)	0.0049
				
Surgery	Resection of primary	69	94 (±6.6)	
	No resection of primary	25	71 (±18)	0.0018
Urinary dopamine	⩽2500 nmol mmol^−1^ creatinine	59	93 (±7.4)	
	>2500 nmol mmol^−1^ creatinine	22	78 (±18)	<0.05
Serum LDH	⩽600 IU l^−1^	12	100	
	>600 IU l^−1^	22	85 (±16.4)	0.23
Serum ferritin	⩽280 ng ml^−1^	30	93 (±9)	
	>280 ng ml^−1^	9	66 (±29)	0.03
Serum NSE	⩽100 nmol ml^−1^	32	100	
	>100 nmol ml^−1^	11	54 (±27.8)	<0.0005
N-myc status	No NMA	59	93 (±6.8)	
	NMA	4	0	<0.0005
1p status	No 1p deletion	17	100	
	1p deletion	6	44 (±38.2)	0.0016

, Figure 2

**Figure 2:**
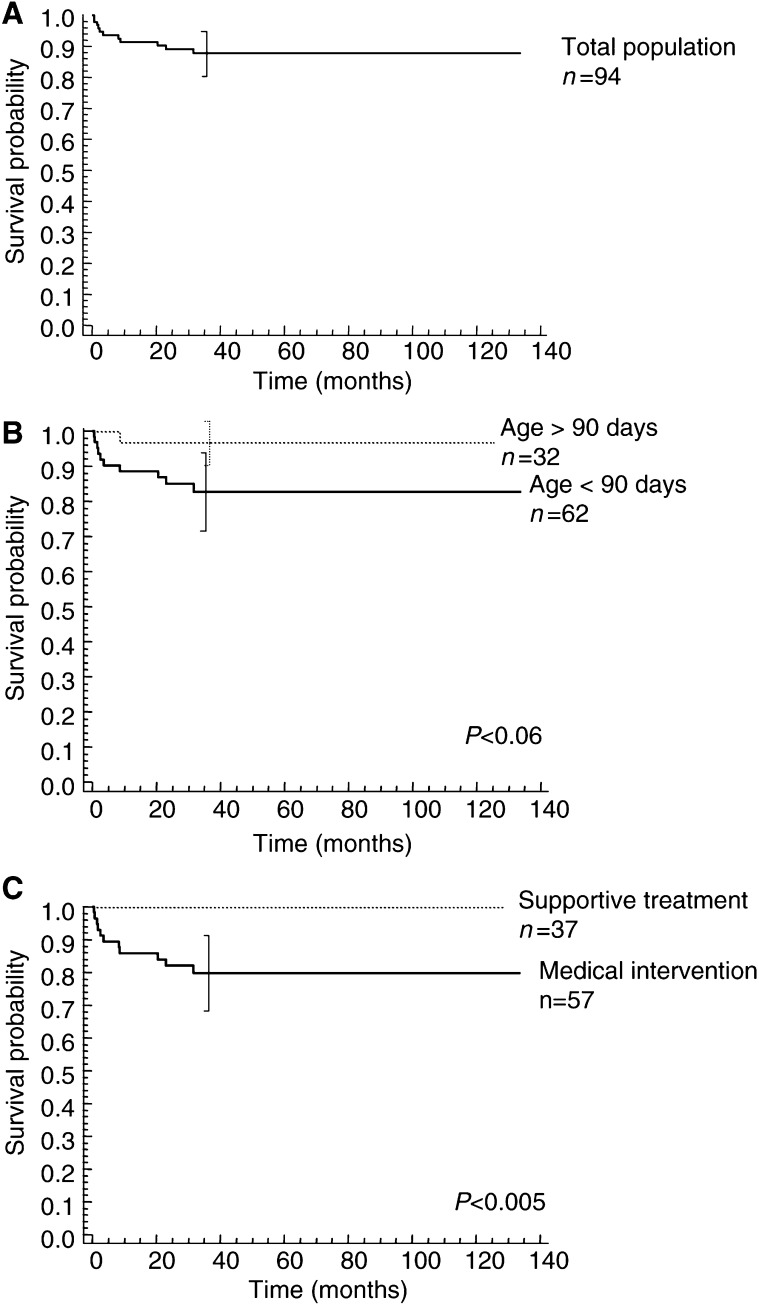
(**A**) Overall survival (OS) of 94 children with stage 4 s neuroblastoma. At 3-year OS (±2SE) was 88% (±7.6). (**B**) OS of 62 infants younger than 3 months at diagnosis, *vs* 32 infants older than 3 months at diagnosis. At 3-year OS (±2 s.e.) was 83% (±12.2) and 93% (±8) in children younger and older than 3 months, respectively (*P*<0.06, log-rank test). (**C**) OS of 37 asymptomatic infants at diagnosis, *vs* 57 infants who required treatment. At 3-year OS (±2 s.e.) was 100 and 80% (±11.8) for those who did not and those who did require treatment, respectively (*P*<0.005, log-rank test).

). No deaths later than 3 years after diagnosis were observed. The site of the primary tumour was not of prognostic significance in this population (Table 3), nor the localisation of metastatic involvement. Although not statistically significant, there was a tendency for infants younger than 3 months at diagnosis to fare worse than those older than 3 months (log-rank test, *P*<0.06). The strongest indicator of poor prognosis was the necessity of medical intervention. Indeed, the OS of the 37 infants who received supportive measures only was 100%, *vs* 79.8% for those who required medical treatment (log-rank test, *P*=0.0049). Thus, those who did require medical intervention fared significantly worse.

### Surgical treatment

Surgical resection of the primary tumour was attempted in the majority of cases (69 out of 94 pts, 73%), with a mean delay of 154 days (range 0–492 days) after diagnosis. Liver metastases were detected peroperatively in three patients leading to a secondary diagnosis of stage 4s NB. For 66 other patients, surgical resection of the primary tumour was performed either after a period of observation (28 out of 37 pts, 75%), or after completing the various primary treatment schemes (38 out of 57 pts, 66%). Parietoplasty was performed additionally in four cases. For 25 patients (27%) surgical resection of the primary tumour was not attempted. In five cases, no primary tumour could be identified. In all, 20 other patients did not undergo resection, seven of whom had failed to respond to medical treatment and died. For the 13 others the decision to refrain from surgery was taken either on account of the primary tumour's localisation or in accordance with the more recent recommendations leaving the decision of surgery to the treating physician. The OS of patients who did not undergo resection of the primary tumour was significantly poorer than that of the patients who were operated (log-rank test, *P*=0.0018, Table 3).

### Biochemistry and molecular biology results

Urinary catecholamines (HVA, VMA, dopamine) and serum LDH, ferritin and NSE levels were determined at diagnosis for 92, 34, 39 and 43 patients, respectively. As shown in Table 3, patients with elevated urinary dopamine, serum ferritin or NSE levels had a worse outcome than those with normal levels. N-myc copy number was evaluated in 63 out of 94 cases (67%), of whom four had NMA. All patients with NMA have died of disease (Table 3). Chromosome 1p status was determined in 23 patients, six of whom had a chromosome 1p deletion, and three of whom have died, also resulting in a significantly worse survival rate (Table 3).

## DISCUSSION

In this retrospective study, we have analysed the outcome of 94 infants presenting with stage 4s NB in SFOP centres between 1990 and 2000. All were classified according to the INSS criteria (Brodeur *et al*, 1993), and bone metastases were excluded in all cases by skeletal surveys (63 out of 94 pts, 67%), MIBG scanning (84 out of 94 pts, 89%) and/or bone scans (14 out of 94 pts, 15%). Strict INSS staging does not include stage 4s NB infants with tumoral bone marrow infiltration exceeding 10%, which has been reported as extremely rare (one patient in 80, Nickerson *et al*, 2000). In our study, the degree of bone marrow involvement was not taken into account.

This study confirms that stage 4s NB is associated with an excellent survival rate. Indeed, the OS is 88% and comparable to previous studies (Suarez *et al*, 1991; De Bernardi *et al*, 1992; Strother *et al*, 1995; Van Noesel *et al*, 1997; Katzenstein *et al*, 1998; Nickerson *et al*, 2000). We confirm that over one-third of patients have clinically favourable tumours without organ dysfunction despite widespread disease, and that for these patients careful observation is sufficient with a survival rate of 100%.

Two-third of patients required medical intervention. However, decisions regarding medical treatment were not taken homogeneously. First, the attitudes regarding large but stable tumours without major organ compromise depended on the investigator. Indeed, in some cases, treatment was initiated for large tumours in the absence of spontaneous regression because of a threat of decompensation. Second, for some patients with PD, a decision to treat was taken after a period of observation, and we have arbitrarily grouped infants for whom this occurred within 60 days following diagnosis with those who were symptomatic and required treatment immediately at diagnosis. This definition may account for the higher percentage of infants requiring initial treatment in our study as compared to other published series (Nickerson *et al*, 2000). Finally, life-threatening symptoms were often defined subjectively according to the treating physicians rather than to objective scoring systems (Hsu *et al*, 1996). These observations underline the necessity of objective criteria for treatment decisions.

Patients with life-threatening symptoms received medical treatment according to several different treatment strategies over the past decade. Radiotherapy as first-line treatment proved unsatisfactory, as it was sufficient treatment in only 20% of patients. Two different first-line chemotherapy schedules were proposed consecutively. The first treatment schedule (CO) also proved to be unsatisfactory as it was sufficient in only 34% of patients, the others receiving either additional radiotherapy and/or second-line chemotherapy with the CE regimen. This regimen used as first-line treatment may be more promising as it was the only treatment given to the six latest infants resulting in a 100% survival rate. Altogether, 12 patients died of disease. Nine of these, including two patients with NMA, presented with relentless disease progression either during first-line treatment (seven pts), or following hepatic recurrence (two pts with NMA). Disease progression was mainly hepatic in eight cases, leading to major organ failure, or pulmonary and hepatic in one case. Three other patients, including two patients with NMA, relapsed as aggressive stage 4 disease. These observations suggest that in patients with rapidly expanding intra-abdominal disease, earlier and more intensive treatment may be necessary.

As already reported, very young infants had a worse prognosis (De Bernardi *et al*, 1992; Katzenstein *et al*, 1998; Nickerson *et al*, 2000). In previous studies, infants less than 2 months at diagnosis were found to fare worse, although in some other studies the threshold was 1 or 3 months old. In our study, the age of 3 months was the most discriminating, and 11 out of 12 patients who died of disease were younger than 3 months at diagnosis.

Surgical resection of the primary tumour in children with stage 4s NB has been recommended in the past, and has been associated with a favourable outcome (Martinez *et al*, 1992). However, such an intervention may also be fraught with significant side effects in small infants, and its systematic application is controversial. In our study, over two-third of all patients underwent surgical resection of the primary tumour, in most cases after a period of observation or after completion of primary medical treatment. Interestingly, those who did not undergo resection of their primary tumour fared worse than those who were operated. These data must be interpreted with caution as they may reflect the fact that in many cases, the absence of surgical resection concerned infants with disease non responsive to medical treatment. Following more recent reports suggesting that resection of the primary tumour does not significantly contribute to outcome, the decision to perform surgery, although recommended, was left to the individual physicians (Guglielmi *et al*, 1996).

Although strict staging according to the INSS criteria does not take into account bilateral tumours, such patients were included in our study if all other INSS criteria were fulfilled (Nickerson *et al*, 2000). Indeed, in 13 of 94 patients (13%) bilateral adrenal disease was demonstrated by imaging studies showing bilaterally enlarged adrenal glands. This incidence is slightly higher than that of 6% reported previously (Nickerson *et al*, 2000). In total, 10 of these patients underwent surgery, either as resection of the larger primary (eight cases), or as complete resection of one and partial resection of the other primary (two cases). After surgery, no local recurrence was observed for any of these patients.

Among the biological and genetic factors studied, NMA was the strongest predictor of a poor outcome. Indeed, all infants who presented NMA died of PD. NMA is rare in stage 4s NB, occurring in less than 10%, but has been strongly associated with a poor outcome in all studies but one (Tonini *et al*, 1997; Van Noesel *et al*, 1997; Katzenstein *et al*, 1998). Our series of patients confirms NMA as an adverse prognostic factor. Other parameters associated with a poor outcome were elevated urinary dopamine excretion as well as elevated serum ferritin and NSE levels, in accordance with previous reports. In our study, chromosome 1p deletion was also associated with a poor outcome. Interestingly, among the 13 patients who died, all but one had at least one unfavourable biological or genetic marker, the patient with only favourable biological and genetic parameters having died of treatment toxicity rather than disease progression. Other biological and genetic factors such as low telomerase activity, low expression of nerve growth factor and its receptor Trk-A, tumour diploidy and gain of chromosome 17q material have been associated with poor outcome in NB (Bown *et al*, 1999). Further studies making use of recent technological advances such as DNA microarrays may allow the identification of factors associated with tumour regression and thus facilitate treatment decisions.

In conclusion, the outcome observed with the different successive treatment approaches suggests that if infants with stage 4s NB do require therapy, a more intensive chemotherapy may be more beneficial. Indeed, in all six patients who received a more intensive treatment by the CE regimen, only two chemotherapy courses were required. Although no randomised trial was performed and although patient numbers are low and follow-up short, the results suggest that a prompt initiation of a more intensive treatment may be necessary in order to push the NB cells towards the regression pathway. If this is achieved early, further intensive treatment may possibly be avoided. In this report, the CE regimen proved to be associated with a high response rate and good clinical tolerance when used as second-line or as first-line therapy, a schedule which has also shown good response rates and clinical tolerance in infants with localised, unresectable NB (Rubie *et al*, 2001). The CE regimen is now being proposed as first-line therapy in the NB99 Infant SIOP study for patients with stage 4s NB who do require medical intervention.
